# Comprehensive analysis of immune implication and prognostic value of DHX33 in sarcoma

**DOI:** 10.1097/MD.0000000000033654

**Published:** 2023-04-28

**Authors:** Xinan Zhang, Yiming Shao, Yaqi Zhou, Zhi Zhu, Xiaohu Wang

**Affiliations:** a Department of Orthopedics, Zhengzhou Central Hospital Affiliated to Zhengzhou University, Zhengzhou, China.

**Keywords:** DHX33, immune infiltration, prognosis, sarcoma

## Abstract

DEAH-box helicase 33 (DHX33) is an RNA helicase that has been identified to promote the progression of a variety of cancers. However, the relationship between DHX33 and sarcoma remains unknown. RNA expression data with clinical information for the sarcoma project was collected from TCGA database. The association between the differential expression of DHX33 and the prognosis for sarcoma was assessed using survival analysis. CIBERSORT was used to evaluate the immune cell infiltration in sarcoma sample tissues. We then further investigated the association between DHX33 and tumor-infiltrating immune cells in sarcoma using the TIMER database. Finally, the immune/cancer-related signaling pathways involved in DHX33 were analyzed using gene set enrichment analysis. High DHX33 expression was discovered to be a poor prognostic indicator in TCGA-SARC. Immune subpopulations in the TCGA-SARC microenvironment are dramatically altered compared to normal tissues. The tumor immune estimation resource analysis revealed a strong correlation between the expression of DHX33 and the abundance of CD8+ T cells and dendritic cells. Changes in copy number also affected neutrophils, macrophages, and CD4+ T cells. According to gene set enrichment analysis, DHX33 may be involved in a number of cancer- and immune-related pathways, such as the JAK/STAT signaling pathway, P53 signaling pathway, chemokine signaling pathway, T cell receptor signaling pathway, complement and coagulation cascades, and cytokine-cytokine receptor interaction. Our study emphasized that DHX33 may be involved in the immune microenvironment of sarcoma and play an important role. As a result, it is possible that DHX33 might serve as an immunotherapeutic target for sarcoma.

## 1. Introduction

Sarcoma is a frequent malignant tumor that is widespread in children and adolescents.^[1]^ Current primary treatment modalities for sarcoma include surgery accompanied by radiotherapy and chemotherapy, which can extend survival of more than 60% of patients without metastases.^[2,3]^ Although it has radically extended the prognosis of sarcoma suffers, clinical outcomes have barely improved over the previous decades.^[4]^ The 5-year overall survival rate for these suffers with recurrence and metastasis is even decrease than 25% due to the emergence of resistance to chemotherapy.^[5]^ Accordingly, further studies on sarcoma are needed to better understand its molecular mechanisms and provide more options for clinical therapeutic strategies to enhance the survival rate of sarcoma patients.

Over the past few years, immunotherapy has made significant advances and become mainstream in a variety of solid tumors.^[6,7]^ Sarcoma was the first tumor model to be suggested for use of immunotherapy as a treatment option.^[8]^ Several clinical studies have demonstrated that immunotherapy has the possibility to improve the results of treatment for patients with osarcoma.^[9,10]^ However, the molecular characterization of the immune microenvironment within the tumor remains to be explored in depth. In order to have a better prognosis, it is critical to discover hub genes from tumor-specific immunophenotypes and investigate the associated mechanisms.

DEAH-box helicase 33 (DHX33) is part of the DEAD/DEAH box protein family and has been discovered to be essential for several facets of RNA metabolism.^[11]^ In previous studies, DHX33 has been defined as a key player in ribosome biogenesis and a cell membrane RNA sensor that activates the NLRP3 inflammasome.^[12,13]^ The DHX33 protein was also found to be dysregulated in several cancers, including lymphoma, glioblastoma, liver cancer, and lung cancer.^[14–17]^ So far, the immunological implication of DHX33 in sarcoma has not been examined and reported. More importantly, the relationship between DHX33 and the prognosis of patients with sarcoma remains to be clarified.

In the present study, we examined the potential value of DHX33 as a prospective target for immunotherapy in sarcoma by evaluating the link between DHX33 and prognosis of patients with sarcoma. In addition, we thoroughly assessed the connections between DHX33 and infiltrating immune cells, as well as DHX33-mediated immune/cancer signaling pathways.

## 2. Materials and methods

### 2.1. Sarcoma gene expression profiles and clinical data

The sarcoma patient data and mRNA transcriptome data were acquired from the The Cancer Genome Atlas (TCGA) database (https://portal.gdc.cancer.gov/). A total of 2 normal sample tissues and 263 sarcoma sample tissues were included. Normalization of all RNA-seq data for subsequent survival analysis.

### 2.2. Differential expression and survival analysis of DHX33 in sarcoma tissues

Boxplots were generated with disease status as a variable to assess the differential expression of DHX33 in different sample tissues (tumor or normal). Expression of DHX33 at a level above or below the median was defined as high or low level, respectively. The relationship between the differential expression of DHX33 and the prognosis of sarcoma patients was evaluated using survival analysis.

### 2.3. Analysis of tumor-infiltrating immune cells in sarcoma

Cell type Identification By Estimating Relative Subsets Of RNA Transcripts (CIBERSORT) was used to compute the relative infiltration ratios of 22 tumor-infiltrating immune cells in all tissues to evaluate the immune cell infiltration in sarcoma sample tissues.^[18]^ R scripts were downloaded from the CIBERSORT website and analyzed for mRNA expression matrices of all samples. Immune infiltrations that may be affected by DHX33 expression were selected based on *P* < .05.

### 2.4. Correlation between DHX33 and tumor-infiltrating immune cells

The Tumor Immune Estimation Resource (TIMER) database contains information on 32 cancers from the TCGA database as well as 10,897 tissue samples (https://cistrome.shinyapps.io/timer/).^[19]^ The “Gene” module was used to assess the relationship between DHX33 expression in sarcoma and the abundance of B cells, CD4+ T cells, CD8+ T cells, neutrophils, macrophages and dendritic cells. Additionally, the “SCNA” module was used to investigate the connection between altered cell copy number and the abundance of immune cell infiltration.

### 2.5. Gene set enrichment analysis

To elucidate significant differences in signaling pathways between DHX33 gene expression levels, c2.cp.kegg.v7.2.symbols.gmt in Gene Set Enrichment Analysis (GSEA) software 4.2.2 (http://www.gsea-msigdb.org/gsea/index.jsp) was used as the control gene set.^[20,21]^ Significant enrichment was defined as normalized enrichment score (NES) > 1, *P* value < .05 and false discovery rate (FDR) < 0.25.

### 2.6. Immunomodulators

TISIDB is a database of tumor-immune interactions containing multiple types of high-throughput data (http://cis.hku.hk/TISIDB/).^[22]^ Immunostimulators and immunoinhibitors obviously associated with DHX33 expression were obtained from TISIDB and further analyzed (Spearman’s correlation test, *P* < .05). The immunomodulators obtained above were uploaded to the STRING online database to construct a protein-protein interaction network.^[23]^ Gene Ontology (GO) functional enrichment analysis and Kyoto Encyclopedia of Genes and Genomes (KEGG) pathway enrichment analysis of immunomodulators were then performed using the WEB-based GEne SeT AnaLysis Toolkit.^[24]^

### 2.7. Statistical analysis

Boxplots and Kaplan–Meier curves were performed and visualized using R software (version 4.1.2) loaded with R package (ggplot2, ggpubr, survival, survminer). Continuous variables were summarized through mean and standard deviations and compared through Student *t* test. The correlation analysis was evaluated in the TIMER database using Spearman’s correlation analysis. The correlations between DHX33 expression and abundance scores of immune cells evaluated by Spearman’s correlation. Results with *P* < .05 were considered as statistically significant, providing credibility for the data analysis.

## 3. Results

### 3.1. Expression levels and survival probability of DHX33 in sarcoma

**Figure 1.**
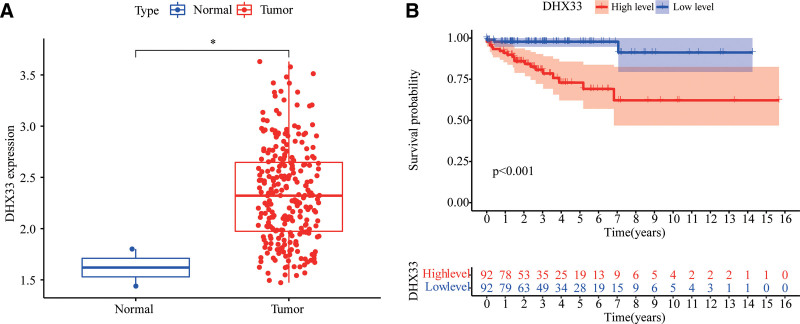
The expression level and survival probability of DHX33 in TCGA-SARC. (A) The expression level of DHX33 in TCGA-SARC. (B) Correlation of differential expression of DHX33 with TCGA-SARC prognosis. TCGA = The Cancer Genome Atlas. *P < .05.

First, we compared the expression of DHX33 in all samples of the TCGA-SARC dataset. We discovered that 263 cancer samples had considerably higher levels of DHX33 expression than did 2 normal samples (Fig. 1A). Second, we compared the survival probability of patients at various DHX33 expression levels. In the DHX33-low group, both the number of survivors and the likelihood of survival were noticeably higher than in the DHX33-high group (Fig. 1B).

### 3.2. The distribution of tumor-infiltrating immune cells in sarcoma

**Figure 2.**
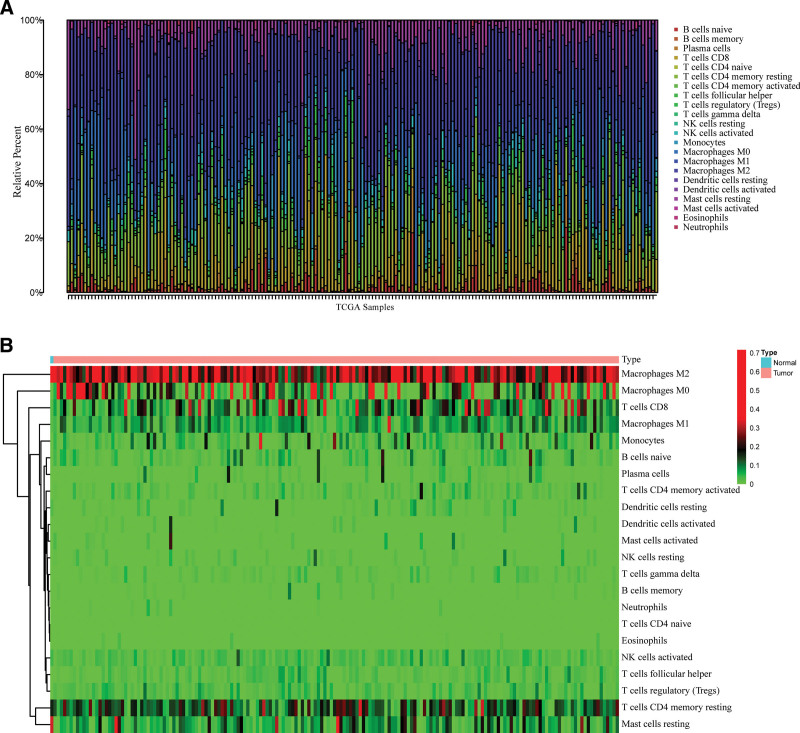
Evaluation of the proportions of 22 types of immune cell infiltration by Cell type Identification by Estimating Relative Subsets Of RNA Transcripts method. (A) The relative percent of different immune cells in each sample. (B) heatmaps indicated the differences in the immune cell distribution between malignant (red) and normal (blue) tissues in SARC cohorts.

Using the CIBERSORT technique, we thoroughly examined 22 immune cell infiltrations in TCGA-SARC samples. In the TCGA-SARC cohort, immune cell infiltration was visualized in cancer and healthy samples after removing samples with *P* ≥ .05 (Fig. 2A). Immune cell differences between tumor and normal tissues were demonstrated using a heat map (Fig. 2B).

### 3.3. Association between DHX33 and tumor-infiltrating immune cells

**Figure 3.**
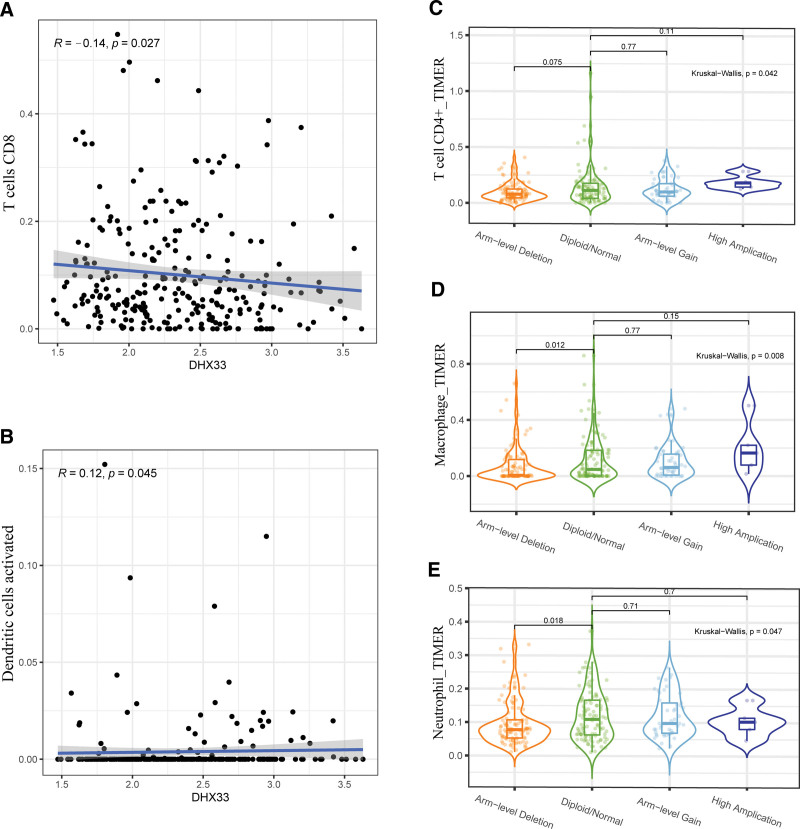
Association between DHX33 and tumor-infiltrating immune cells. (A–B) Correlation between DHX33 expression and the abundance of immune infiltration. (C–E) Associations between DHX33 gene copy numbers and immune cell infiltration levels.

Next, we explored whether the expression level of DHX33 correlated with immune cell in sarcoma. The findings revealed a negative correlation between DHX33 and CD8+ T cells and a positive correlation between DHX33 with dendritic cells (Fig. 3A and B). In addition, we discovered that the infiltration of CD4+ T cells, macrophages and neutrophils in SARC were associated with alterations in DHX33 gene copy number (Fig. 3C–E).

**Figure 4.**
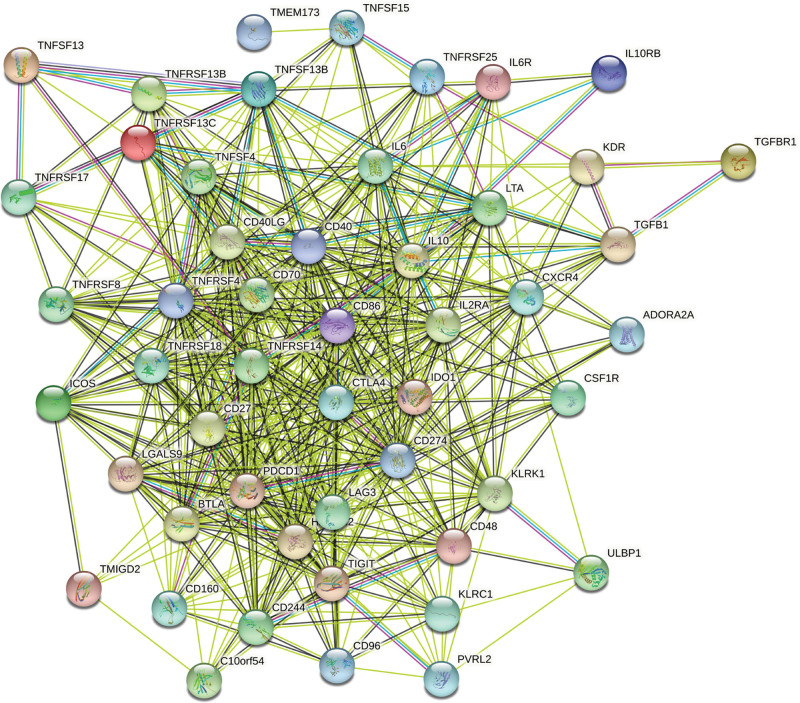
PPI network of 50 DHX33-associated immunomodulators in sarcoma. PPI = protein–protein interaction.

**Figure 5.**
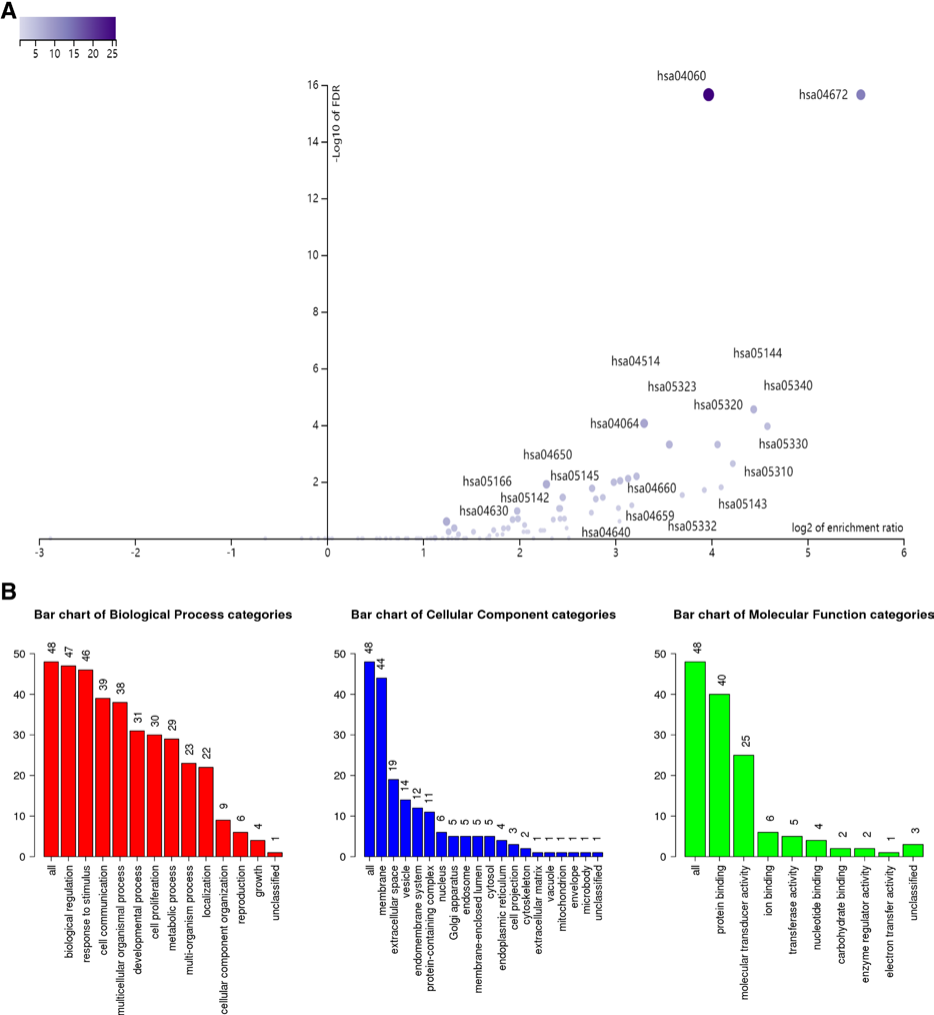
Analysis of immunomodulators associated with the DHX33. (A) KEGG signaling pathway analysis. (B) GO annotation. GO = gene ontology, KEGG = Kyoto Encyclopedia of Genes and Genomes.

Subsequently, we also examined the immunomodulatory role of DHX33 in the development of sarcoma. We found that the expression of DHX33 in sarcoma was substantially correlated with 30 immunostimulators and 20 immunoinhibitors (Table 1). A protein-protein interaction network was developed in order to highlight the relationships between these immunomodulators (Fig. 4). The KEGG signaling pathway enrichment analysis of these genes revealed that DHX33-mediated immune regulation involves the NF-B signaling pathway, T cell receptor signaling pathway, natural killer cell-mediated cytotoxicity, and JAT/STAT signaling pathway (Fig. 5A; Table 2). These immunomodulators’ GO functional enrichment analysis results were also shown visually (Fig. 5B).

**Table 1:** Immunomodulators associated with DHX33 in sarcoma.

Immunostimulators	Immunoinhibitors
C10orf54, CD27, CD40, CD40LG, CD48, CD70, CD86, CXCR4, ICOS, IL2RA, IL6, IL6R, KLRC1, KLRK1, LTA, TMEM173, TMIGD2, TNFRSF4, TNFRSF8, TNFRSF13B, TNFRSF13C, TNFRSF14, TNFRSF17, TNFRSF18, TNFRSF25, TNFSF4, TNFSF13, TNFSF13B, TNFSF15, and ULBP1	ADORA2A, BTLA, CD96, CD160, CD244, CD274, CSF1R, CTLA4, HAVCR2, IDO1, IL10, IL10RB, KDR, LAG3, LGALS9, PDCD1, PVRL2, TGFB1, TGFBR1, and TIGIT

**Table 2:** The results of KEGG pathway enrichment analysis.

Term	Count	*P* value
hsa04060 Cytokine-cytokine receptor interaction	26	0
hsa04672 Intestinal immune network for IgA production	13	0
hsa05144 Malaria	6	2.57E-07
hsa04514 Cell adhesion molecules (CAMs)	8	1.07E-06
hsa05340 Primary immunodeficiency	5	1.69E-06
hsa05323 Rheumatoid arthritis	6	9.65E-06
hsa05320 Autoimmune thyroid disease	5	1.04E-05
hsa05330 Allograft rejection	4	5.59E-05
hsa04064 NF-kappa B signaling pathway	5	1.77E-04
hsa04660 T cell receptor signaling pathway	5	2.36E-04
hsa04659 Th17 cell differentiation	5	3.09E-04
hsa05145 Toxoplasmosis	5	3.82E-04
hsa05166 Human T-cell leukemia virus 1 infection	7	4.86E-04
hsa05310 Asthma	3	6.70E-04
hsa04650 Natural killer cell mediated cytotoxicity	5	7.83E-04
hsa05143 African trypanosomiasis	3	9.61E-04
hsa05332 Graft-versus-host disease	3	.001529008
hsa04630 JAK-STAT signaling pathway	5	.002022723
hsa04640 Hematopoietic cell lineage	4	.002065304
hsa05142 Chagas disease (American trypanosomiasis)	4	.002482216

KEGG = Kyoto Encyclopedia of Genes and Genomes.

### 3.4. DHX33 involved in various immune/cancer-related pathways of sarcoma

**Figure 6.**
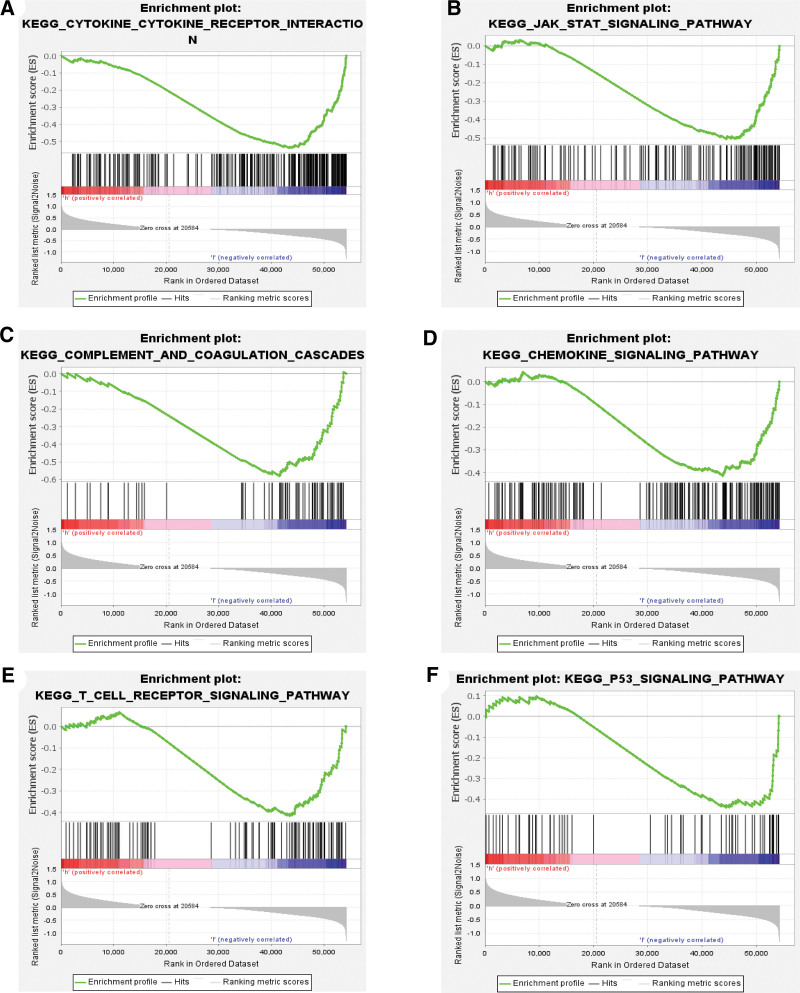
Dissection of DHX33-associated immune signaling pathways by Gene Set Enrichment Analysis. (A–F) DHX33 is associated with cytokine–cytokine receptor interaction, JAK/STAT signaling pathway, complement and coagulation cascades, chemokine signaling pathway, T cell receptor signaling pathway, and P53 signaling pathway. JAK = Janus Kinase, STAT = signal transducer and activator of transcription.

To investigate the function of DHX33 in the immune microenvironment of sarcoma, we used GSEA analysis to probe into the pertinent signaling pathways in which DHX33 is involved. GSEA analysis indicated that DHX33 is involved in various immune/cancer-related signaling pathways, including cytokine–cytokine receptor interaction, JAK/STAT signaling pathway, complement and coagulation cascades, chemokine signaling pathway, T cell receptor signaling pathway, and P53 signaling pathway (Fig. 6A–F). These findings strongly suggest that DHX33 may participate in the initiation and development of sarcoma via the associated pathways mentioned above.

## 4. Discussion and conclusion

Due to the highly aggressive nature of sarcoma, early distant metastasis and recurrence are currently the major issues limiting treatment outcomes and survival rates. Therefore, the development of new therapeutic approaches is crucial. In recent years, immunotherapies have sprung up and brought hope to the treatment of sarcoma. Immunotherapy has great potential and is well recognized in the treatment of sarcoma.^[25,26]^ Exploring key molecular markers that accurately reflect a patient’s immune status and prognosis will be of great value in therapeutic decision-making for sarcoma.

Recent studies have shown that DHX33 is involved in cancer progression and serves as a potential diagnostic marker for certain cancers, including colon cancer and glioblastoma.^[15,27]^ However, up to date, neither the expression of DHX33 in sarcoma patients nor its effect on the immune microenvironment has been reported. We found that the expression of DHX33 was significantly higher in TCGA-SARC tissues compared to normal tissues, indicating that DHX33 may play a role in the ability of sarcoma to promote tumor growth. Most importantly, the worsening prognosis of sarcoma was associated with the overexpression of DHX33. We also found dramatic changes in immune subsets in the TCGA-SARC microenvironment compared to normal tissues. The above results suggest that it may be feasible to predict the overall survival of patients with sarcoma by indicating their immune status by DHX33.

Therefore, we further determined the impact of DHX33 on immune cell infiltration in sarcoma. The expression of DHX33 was strongly related to the abundance of CD8+ T cells and dendritic cells. Copy number changes also resulted in altered CD4+ T cells, macrophages and neutrophils. We used GSEA analysis to investigate the pertinent enrichment pathways to gain more insight into the potential mechanisms of DHX33 in sarcoma. These findings significantly support the hypothesis that DHX33 participates in a number of immune-related pathways, including the cytokine-cytokine receptor interaction, the T cell receptor signaling pathway, the complement and coagulation cascades, and the chemokine signaling pathway. Apoptosis, the JAK/STAT signaling pathway, the P53 signaling pathway, and other cancer-related pathways were also enriched.

Janus kinase (JAK) is a non-receptor type protein tyrosine kinase that is essential in the signaling cascade of cytokines. When JAK is phosphorylated, it is able to activate signal transducers and activators of transcription (STAT) to dimerize.^[28]^ Mesenchymal stem cells activate the JAK/STAT signaling pathway by inducing IL-6 expression and binding to homologous receptors in osteosarcoma cells.^[29]^ P53 is an important tumor suppressor of which mutation drive tumorgenesis/cancer development.^[30]^ Early investigations revealed that osteosarcoma typically had p53 mutations, and following research examined the clinical importance of p53 mutations or p53 protein overexpression in osteosarcoma.^[31,32]^ A meta-analysis conducted by Pakos et al^[33]^ concluded that P53 alterations might be associated with poor survival in patients with osteosarcoma. DHX33-associated immunomodulators were enriched to the classical NF-κB signaling pathway. Proteins that regulate NF-κB signaling are altered or expressed abnormally during tumorigenesis, which leads to improper communication between malignant cells and other organisms. This mechanism has been implicated in osteosarcoma cell proliferation and differentiation.^[34]^ In addition, the production of pro-inflammatory and pro-angiogenic cytokines around osteosarcoma cells can also be controlled by NF-κB.^[35]^

Even though our results imply that DHX33 may be used as a prognostic biomarker for sarcoma, there are still a number of restrictions. First, bioinformatics techniques based on open-access databases were mostly used to carry out our study. This only serves a predictive role and further experimental validation is needed. Second, further studies should be conducted in the future on the possible mechanism and prognostic usefulness of DHX33 in sarcoma.

In conclusion, we discovered that poor prognosis in sarcoma was strongly correlated with increased expression of DHX33. DHX33 plays an important role in the immune microenvironment of sarcoma and is involved in multiple immune/cancer signaling pathways. However, more studies are needed to fully understand the complex mechanisms of DHX33 in the immunomodulatory process in sarcoma.

## Author contributions

**Conceptualization:** Xinan Zhang, Yiming Shao, Xiaohu Wang.

**Data curation:** Xinan Zhang, Yiming Shao, Yaqi Zhou.

**Formal analysis:** Xinan Zhang, Yiming Shao, Yaqi Zhou.

**Funding acquisition:** Zhi Zhu.

**Methodology:** Xinan Zhang, Zhi Zhu, Xiaohu Wang.

**Supervision:** Xiaohu Wang.

**Validation:** Xiaohu Wang.

**Visualization:** Xinan Zhang, Yiming Shao.

**Writing – original draft:** Xinan Zhang.

**Writing – review & editing:** Xiaohu Wang.
